# Supplementary material for: Diagnostic Accuracy of the Deep Learning Model for the Detection of ST Elevation Myocardial Infarction on Electrocardiogram
Source: J Pers Med. 2022 Feb 23;12(3):336. doi: 10.3390/jpm12030336 (PMC8956114; doi:10.3390/jpm12030336)
Supplement: Supplementary file 1 [file jpm-12-00336-s001.zip › supplement Figure S1.pdf]

Supplementary Figure S1. The 12-lead electrocardiogram and heatmap images of false positive cases by the deep learning model.

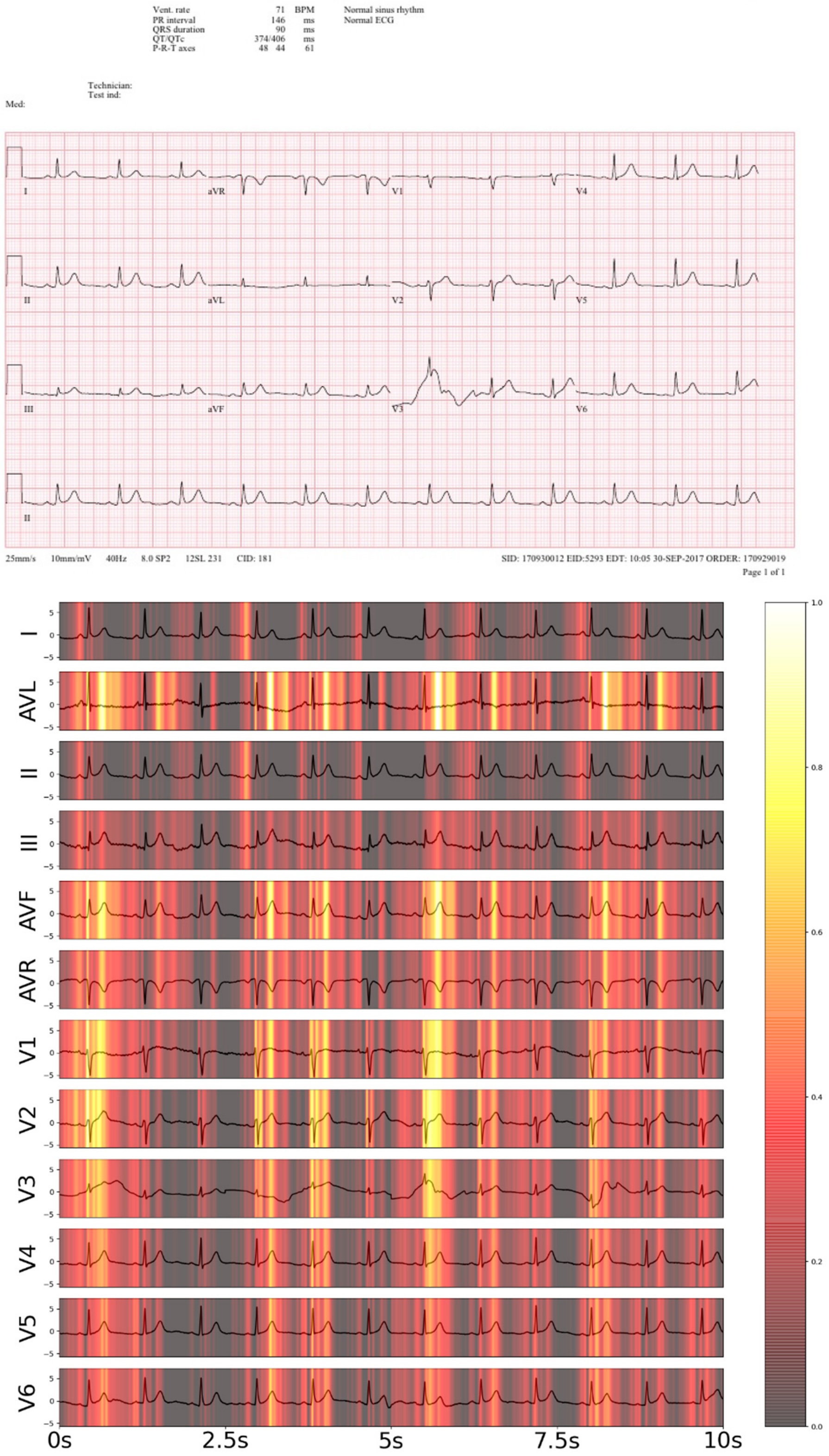

(A) Case 1. In V3 lead, there's a baseline wander with a pointed shape that may be mistaken for ST elevation.

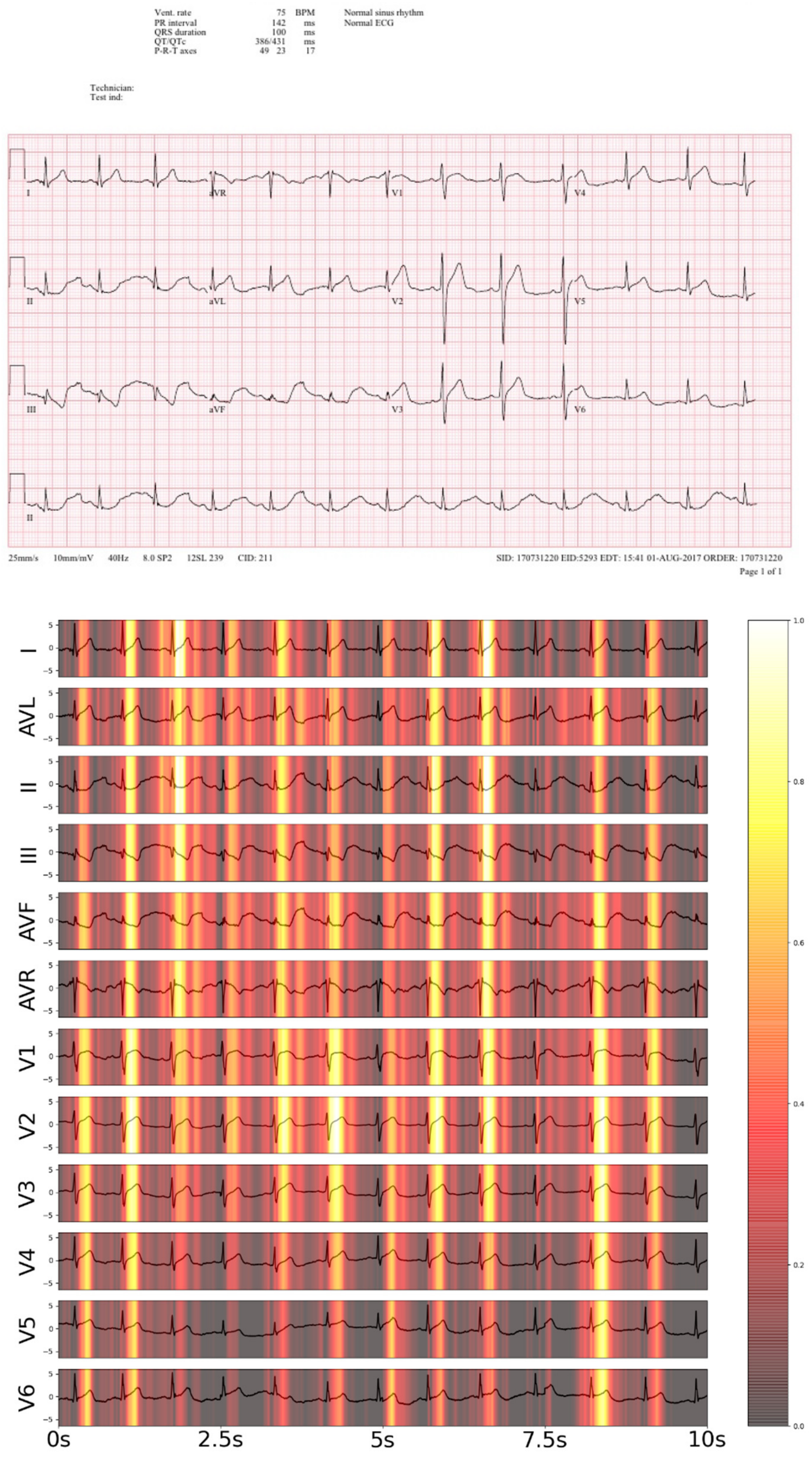

(B) Case 2. Widespread baseline wander in limb leads.

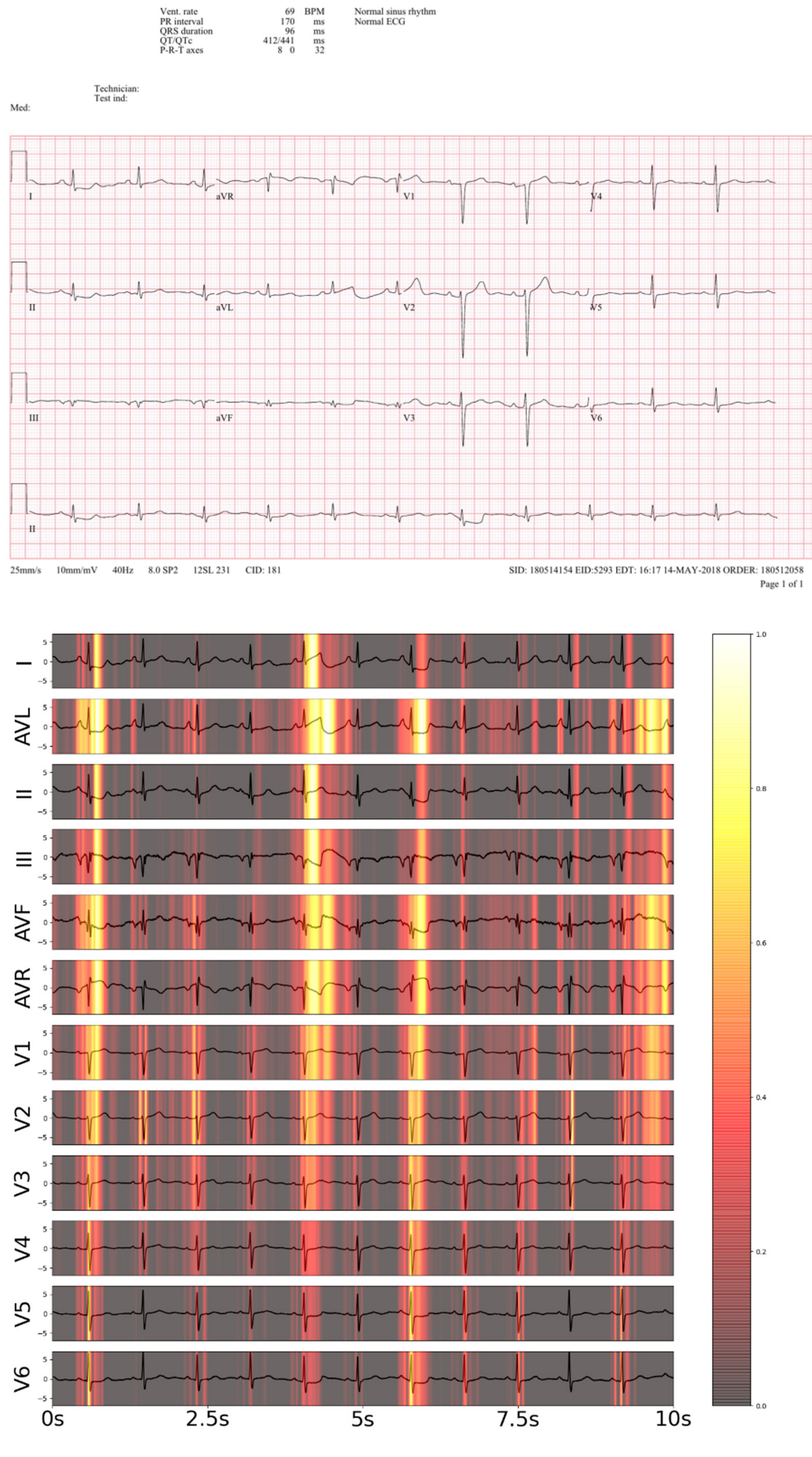

(C) Case 3. Baseline wander causes a misinterpretation of ST elevation in several leads.

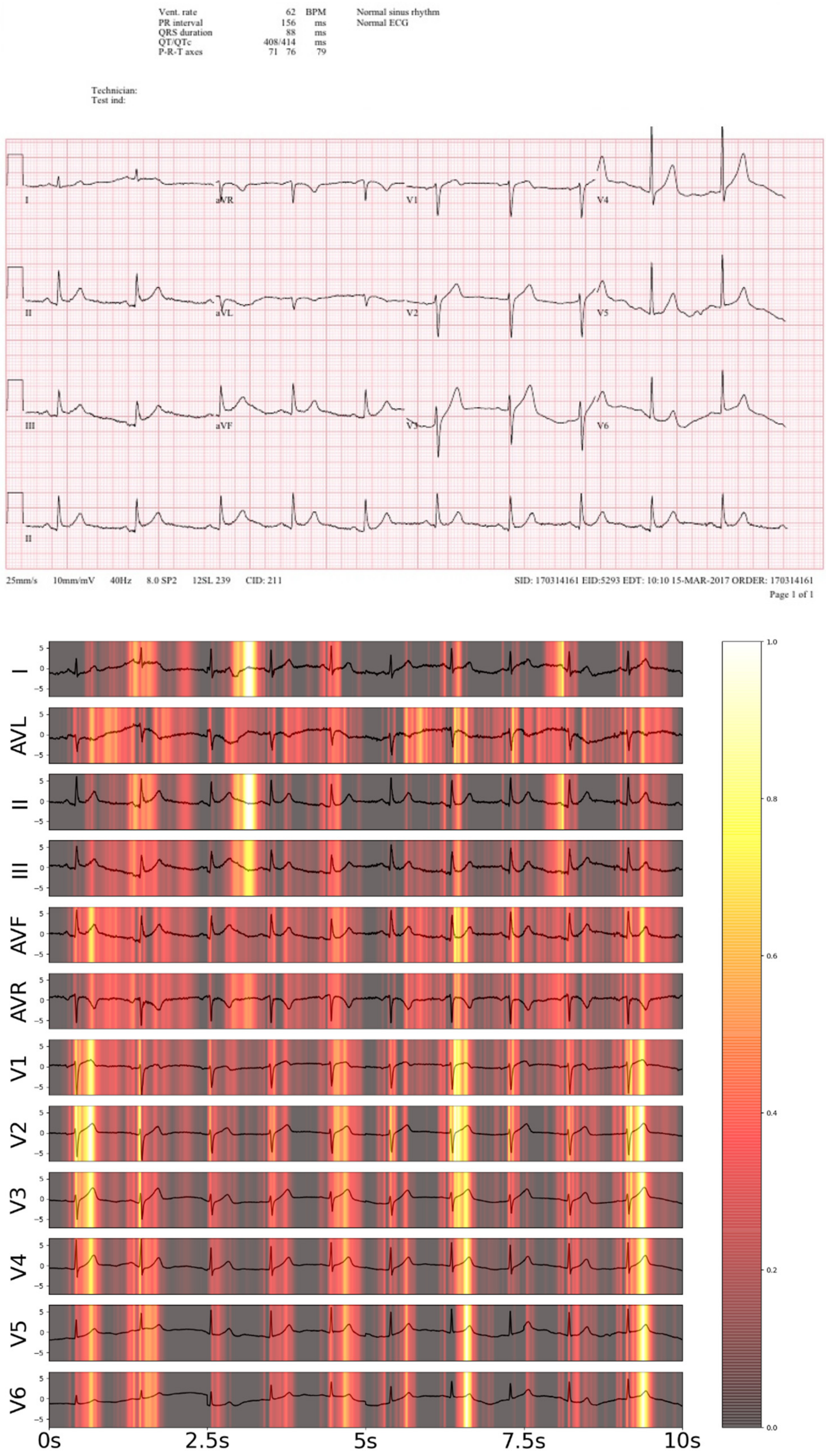

(D) Case 4. Baseline wander causes a misinterpretation of ST elevation in several leads.

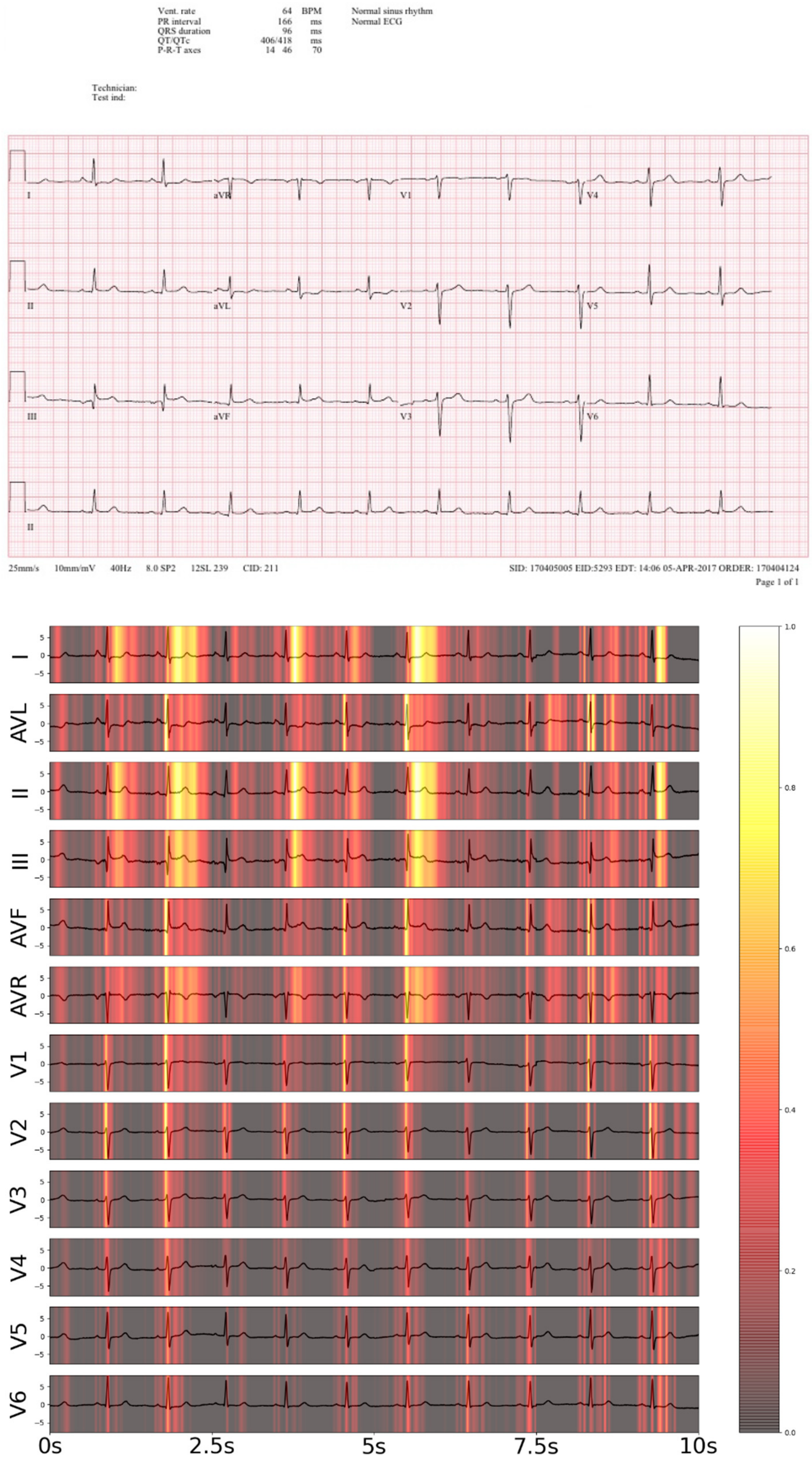

(E) Case 5. A deep learning model detected true ST elevation myocardial infarction with total occlusion of the right coronary artery.

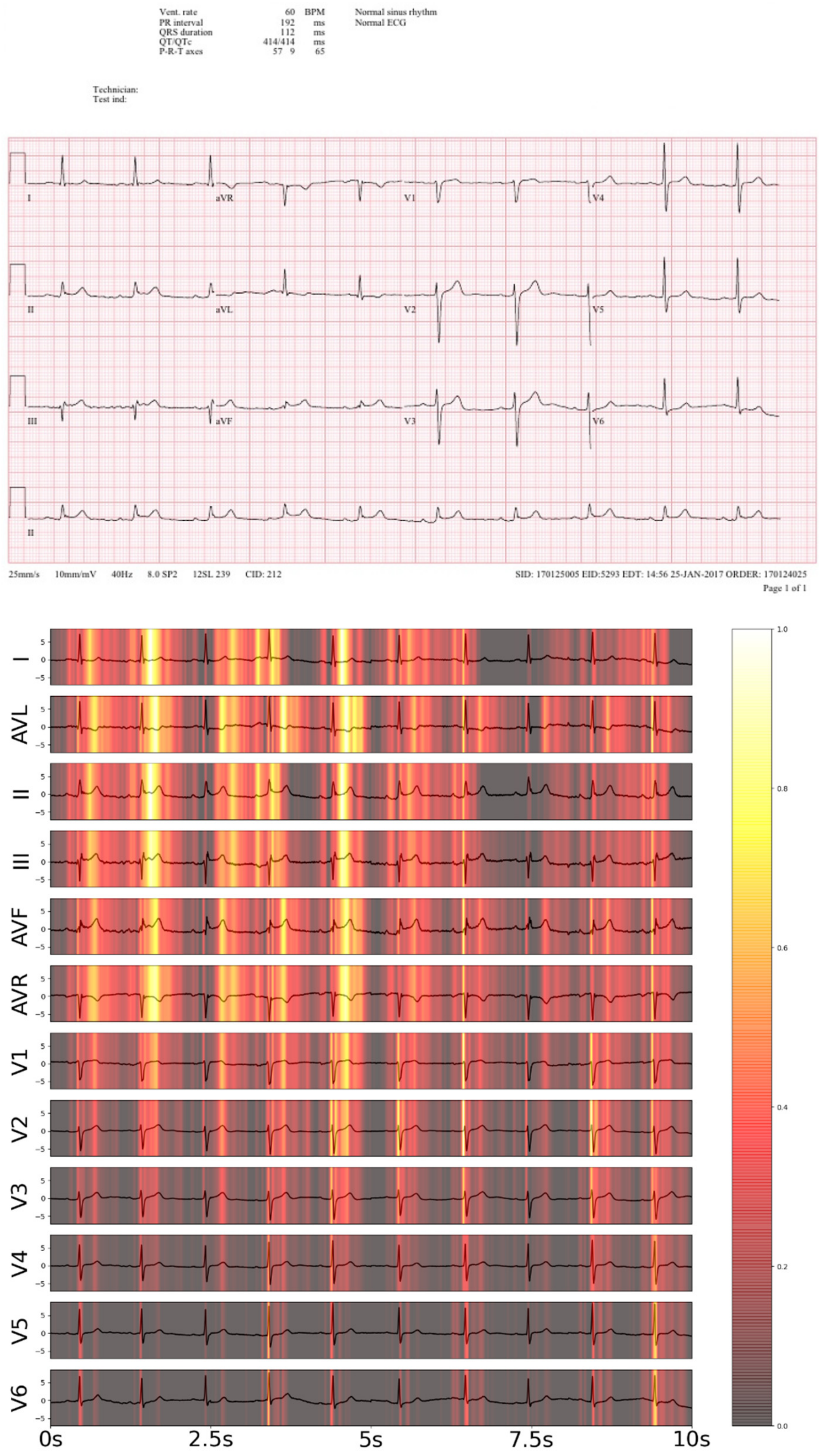

(F) Case 6. A deep learning model detected true ST elevation myocardial infarction with total occlusion of the right coronary artery.
