# Supplementary material for: Diagnostic Accuracy of the Deep Learning Model for the Detection of ST Elevation Myocardial Infarction on Electrocardiogram
Source: J Pers Med. 2022 Feb 23;12(3):336. doi: 10.3390/jpm12030336 (PMC8956114; doi:10.3390/jpm12030336)
Supplement: Supplementary file 1 [file jpm-12-00336-s001.zip › supplementary Document S1.pdf]

Supplementary Document S1

1. Deep Learning Model Architecture details

- The model was based on the Resnet structure [15] and there were some modifications.
- The input was changed into 1D (ECG signal) CNN from 2D (image) CNN.
- The first encoder block is consisted of of Batch normalization layer, ReLU activation layer, followed by Convolutional layer (filter size = 16, stride = 1)
- Next, the encoder has 16 residual blocks. The channel size is doubled at every 4 blocks. Start from 64 -> 128 -> 256 -> 512.
- each block is consisted of two set of convolution layers (batch norm, ReLU, convolution). Followed by the skip connection with max pooling between the beginning and end of the block to make the residual block.
- The filter size and stride for each block was following:
  - Block1-3: (16, 1), Block4: (16, 2)
  - Block5-7: (2, 1), Block8: (2, 2),
  - Block9-11: (4, 1), Block12: (4, 2)
  - Block13-15: (8, 1), Block16: (8, 2)
- Take average pooling by channel wise
- Each 12 weight shared encoders output 512 values and they are concatenated to 6144
- Fully connected layer 6144 -> 1 (Sigmoid)
- Output the probability of STEMI (near "1") or Others (near "0")
